# A Seinhorst Model Determined the Host-Parasite Relationships of *Meloidogyne javanica* Infecting Fenugreek cv. UM202

**DOI:** 10.2478/jofnem-2023-0005

**Published:** 2023-02-28

**Authors:** Hera Nadeem, Amir Khan, Rishil Gupta, Arshi Anees, Faheem Ahmad

**Affiliations:** Department of Botany, Faculty of Life Sciences, Aligarh Muslim University, Aligarh202002, India; Centre for Agricultural Education, Faculty of Agricultural Sciences, Aligarh Muslim University, Aligarh202002, India.

**Keywords:** Fenugreek, Reproduction factor, Root-knot nematode, Threshold level, Host-parasitic relationship, *Meloidogyne javanica*

## Abstract

Root-knot nematodes (RKNs) have been shown to be challenging and persistent pests of economic crops worldwide. Among RKNs, *Meloidogyne javanica* is particularly important, as it rapidly spreads and has a diverse host range. Measuring its damaging threshold level will help us to develop management strategies for adequate plant protection against nematodes. In our study, we observed the relationship between a linear series of 12 initial population densities (P_i_) of *M. javanica*, i.e., 0, 0.125, 0.25, 0.5, 1, 2, 4, 8, 16, 32, 64, and 128 second-staged juveniles (J2s) g^-1^ soil, and fenugreek cv. UM202 growth parameters were investigated using a Seinhorst model. A Seinhorst model was fitted to shoot length and dry weight data for fenugreek plants. A positive correlation was found between J2s inoculum levels and percent reductions in growth parameters. The 1.3 J2s of *M. javanica* g^-1^ soil were found to damage threshold levels with respect to shoot length and shoot dry weight of fenugreek plants. The minimum relative values (m) for shoot length and shoot dry weight were 0.15 and 0.17, respectively, at P_i_ =128 J2s g^-1^ soil. The maximum nematode reproduction rate (P_f_ /P_i_) was 31.6 at an initial population density (P_i_) of 2 J2s g^-1^ soil.

Fenugreek (*Trigonella foenum-graecum* L.) is an annual plant. The leaves and seeds of fenugreek are essential components of our daily diet and are known as functional foods due to their excellent nutritional, antioxidant, and anti-cancer properties (Das et al., 2016). India leads fenugreek production worldwide with an area of 126,294 ha, production of 182,170 tonnes, and average productivity of 1,012 kg ha^-1^ as of 2019-20 (Spice Board of India, 2021). However, the agricultural and environmental conditions that enhance the development of fungal, bacterial, viral, nematode, and insect-pest diseases substantially restrict crop yield (Aljerf, 2018). Plant-parasitic nematodes (PPNs) such as *Helicotylenchus indicus, Tylenchorhynchus brassicae*, *Rotylenchulus reniformis*, and *Meloidogyne incognita* have been found to be associated with fenugreek (Khare et al., 2014). The detrimental effect of nematodes on fenugreek plants is broadly accepted (Kumar and Pathak, 2004). RKNs are considered the most destructive pests, causing huge losses in vegetable crops worldwide and influencing the widespread use of agrochemicals (Sikandar et al., 2020). It has been estimated that PPNs reduce crop yield by approximately 8.8% in advanced countries and 14.6% in tropical and subtropical regions (Mitiku, 2018). RKNs are endo-parasites of roots and have been assessed to cause around $173 billion of annual damages to crops planted worldwide (Gamalero and Glick, 2020). Three components have been used to determine yield losses: i) *Meloidogyne* species and “race,” ii) nematode population density, and iii) host plant species and cultivar (Janati et al., 2018). Post-penetration, they colonize within the roots, affecting water and nutrient intake and hindering mineral translocation. These changes harm plant health, resulting in inadequate development (Gullino et al., 2019). In addition, RKNs alter physiological and metabolic functions, such as photosynthesis in plants, along with their physical attributes (Chen et al., 2022). RKNs have complex interactions with their hosts and the plant genes that are induced during a compatible plant–nematode interaction. A large number of the genes that are induced by infection are likely to contribute to establishing the parasitic interaction (Gheysen and Fenoll, 2022; Puthoff et al., 2003). The extensive changes in cell-wall architecture occur during the development of giant cells, and nematode infection up-regulates genes that encode host cell-wall degrading enzymes. Siddique et al. (2015) reported that nematode-derived cytokinin activates the host cell cycle during infection. These nematodes can synthesize and secrete cytokinin, a functional plant hormone, to establish long-term parasitism. The juvenile RKNs move intercellularly after penetrating the root, migrating down the plant cortex towards the root tip. The juveniles (J2s) then enter the base of the vascular cylinder and migrate up the root (Wyss et al., 1992). They establish a permanent feeding site in the differentiation zone of the host plant’s root.

Damage caused to plants by RKNs is influenced by nematode species as well as the initial nematode population density in soil (Archidona-Yuste et al., 2018). The distribution of these RKN species in field and nurseries are related to abiotic and biotic factors (Hamza et al., 2017). Abiotic factors such as pH, soil type, organic matter content, moisture (Norton, 1978), and local climatic conditions affect nematode development (Neher, 2010). They move only short distances and, thus, their dissemination is via water (Villenave et al., 2003) and wind (Orr and Newton, 1971). Human activities such as the introduction of infected planting material or diffusion of infested soil with nursery practices also contribute to spreading (Alenda et al., 2014). A recent study was conducted by Hamza et al. (2017) on the diversity of RKNs in Moroccan olive nurseries and orchards, analyzing *M. javanica* dispersion based on invasion processes. They reported that the majority of *M. javanica* phenotype J3s were detected in nurseries compared to the orchards and were widespread throughout the main olive-producing areas. Thus, a better understanding of the soil’s biology and initial nematode population density during sowing time is critical for establishing more environmentally safe control measures (Evlice et al., 2022). The damage threshold level is the nematode density in the soil at which yield starts to decline considerably (Korayem et al., 2015). It is an important concern before sowing, to escape unforeseen crop damages, and before implementing extreme synthetic/control measures. Researchers have now developed specific models to predict the damage caused by nematodes in various crops (Seinhorst, 1965, 1979; Viaene et al., 1997). It is widely documented that population density causes the magnitude of crop damage induced by nematodes in the soil at the time of sowing, and also a minimum population density (T) is needed before noticeable yield loss starts (tolerance limit) (Vovlas et al., 2008). It could be as low as 0.25 eggs and second-staged juveniles per cm^3^ soil for susceptible crops such as spinach (Di-Vito et al., 2004). However, no evidence supporting a precise RKN damage threshold has been established for fenugreek, despite India leading fenugreek production worldwide and exporting it to about 84 countries around the globe (Meena et al., 2019). Therefore, this study aims to determine the relationships between the population density of *M. javanica* and fenugreek cv. UM202 growth. The tolerance limit (threshold level) was measured using the Seinhorst model.

## Materials and Methods

*Nematode inoculum*: The RKNs *Meloidogyne javanica* were used in our study. The egg mass was extracted from infected susceptible eggplant “BR-112” roots. The *M. javanica* culture was established and maintained from a single egg mass of an adult female. The method proposed by Hussey and Barker (1973) was followed to obtain the egg suspension. The infected two-month-old eggplant roots were harvested and macerated with 1% sodium hypochlorite for five minutes. The obtained egg suspension was strained through a 25 μm pore size sieve and positioned in Baermann trays. Every 2 hr, hatched second-staged juveniles (J2s) were collected, and fresh tap water was poured. This process was repeated for up to three days to get J2s of *M. javanica*. The obtained J2s were used as the inoculum for the experiment.

*Identification of root-knot nematode, M. javanica*: Adult females of *M. javanica* were extracted from the root galls of infected root samples using dissecting needles. A single mature female was placed on a glass slide, delicately teased at the neck, and squeezed from the posterior side to remove body content. The remaining part was kept in cold lactophenol with 0.03% cotton blue for 24 hr at 28 ± 2°C. The female’s posterior part was prudently cut with a sharp scalpel and trimmed around the perineal pattern. The inner tissue was carefully cleaned in order to see a clear picture, and the perineal pattern was mounted for observation.

*Scanning Electron Microscopy (SEM) of perineal pattern*: The SEM technique was employed to visualize the perineal pattern better. Approximately 10 neatly cut perineal patterns with the surface upwards were transferred to a small quantity of 45% lactic acid on a round coverslip (13 mm diameter) and bordered with glyceel to form a chamber. The cover slip was fixed to the microscopic slide using glyceel on the edges. After 2–3 min, a drop of 2% formalin was added to the chamber to wash out the lactic acid. 10 min later, with the help of blotting paper, excess solution was absorbed, and the perineal pattern was allowed to dry in a desiccator at 28 ± 2°C (Abrantes and Santos, 1989). Before analysing in the SEM (JSM 6510LV; Jeol, Tokyo, Japan), the coverslip was cautiously removed from the microscope slide and attached to the adhesive side of the SEM stub and gold coated (thickness~14 nm). The obtained SEM images of the surface morphology of the perineal pattern were used to identify the *Meloidogyne* spp. as Eisenback (1985) described.

*Effect of initial densities of M. javanica on fenugreek*: A pot experiment was performed to demonstrate the pathogenicity and determine the threshold level of *M. javanica* on fenugreek cv. UM202. A sufficient quantity of freshly prepared J2s inoculum was combined with a potting mix of autoclave-sterilized soil (fine-grained silts and clays soil) and organic manure (3:1) and filled in 1 kg clay pots to achieve a range of increasing population densities of 0, 0.125, 0.25, 0.5, 1, 2, 4, 8, 16, 32, 64 and 128 J2 g^-1^ soil. Fenugreek cv. UM202 seeds were surface-sterilized by dipping in 95% (v/v) ethanol for 10 s, followed by a 3 min treatment with 0.5 % (v/v) sodium hypochlorite, and then washed thrice with distilled water. Seeds were soaked overnight and later sown into each pot. The pots were kept in the glasshouse with four replicates of each nematode population density in a completely randomized block design. The moisture in pots was maintained throughout the experiment. The symptoms caused by *M. javanica* were seen on the fenugreek plant during the investigation. The experiment was terminated at 90 days after sowing, the harvested roots were thoroughly washed to remove the adhered soil, and the morphological parameters (shoot length and dry weight) and pathological parameters (reproduction factor and root-knot index) were recorded. The experiment was repeated twice.

*Estimation of nematode population*: To estimate the final nematode population (P_f_) in each pot, the soil of each population density was appropriately blended, and 100 g of a sub-sample was sieved, as suggested by Cobb’s sieving and decanting method, followed by the Baermann funnel technique (Southey, 1986). The nematode suspension obtained from the soil sample was collected in a beaker and diluted to 100 mL. The suspension was bubbled with a sterilized pipette to facilitate a uniform nematode distribution, and 10 mL of it was transferred to a counting dish. The number of J2s was counted under the stereo microscope (Leitz Wetzlar Vintage, Germany) with three replicates for each treatment. The average of three such counts was taken, and the population density of *M. javanica* g^-1^ soil was estimated. The reproduction rate (P_f_/P_i_) at each initial nematode population density (P_i_) was also determined.

*Root-knot Index (RKI)*: The extent of root-knot infestation was assessed as the root-knot index (RKI), where 0 = no galling, 1 = 1-2 galls, 2 = 3-10 galls, 3 = 11-30 galls, 4 = 30-100 galls and 5 = >100 galls (Taylor and Sasser, 1978).

*Statistical analysis*: The relationship between the initial nematode population density (P_i_) and plant growth (estimated by the shoot length and dry weight) was determined by fitting the data to the Seinhorst model: y = m + (1 – m) z ^Pi−T^ when P_i_ ≥ T, and y = 1 when P_i_ < T (Seinhorst, 1965, 1979). In this model, y is the ratio of the estimated variable for plant growth in an initial population density of the nematode (P_i_), divided by the value obtained in the control plant; m is the minimum yield at a very large initial population density of nematode; P_i_ is the initial nematode population density; T is the tolerance limit; and z is a constant < 1 reflecting nematode damage (Seinhorst, 1965, 1979). For the Seinhorst analysis, we used the SeinFit software, DOS version, developed by Viaene et al. (1997). The coefficient of determination (r^2^) and the residual sum of squares was used to indicate the goodness-of-fit of data to the model. Statistical data analysis of the data was carried out with the open-source software R Version 2.14.1. The significance of differences among treatments was determined by Duncan’s multiple range test (DMRT). According to DMRT at *P* ≤ 0.05, means depicted by the same letter are not significantly different.

## Results

**Figure 1:**
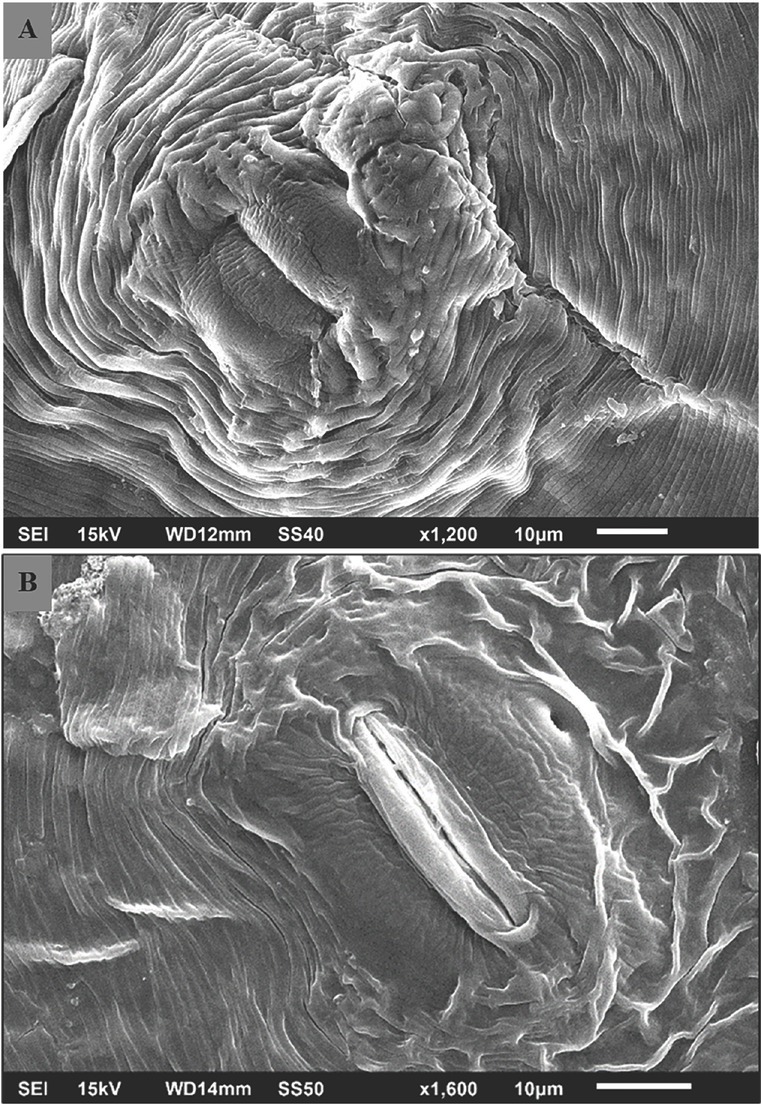
Scanning electron microscopy (SEM) images of the perineal pattern of *M. javanica*, which show a rounded to flattened dorsal arch and conspicuous lateral lines that separate the dorsal and ventral regions of the patterns. (A) A close view of the distinct lateral line in a perineal pattern distinguishes this species from other *Meloidogyne* spp. (B) An inner area was marked by coarsely broken striae and contained the vulva and anus.

*Identification of M. javanica based on the perineal pattern*: Perineal pattern is undoubtedly the most important morphological feature used for the primary identification of RKN species. SEM (Figs. 1A,B) of the perineal pattern shows a rounded to flattened dorsal arch. The characteristic features of this pattern were the distinct lateral incisures that separated the pattern into dorsal and ventral regions. Some striae crossed the lateral incisures, while some bent towards the vulva. The morphological characteristics described the species as *M. javanica*.

**Figure 2:**
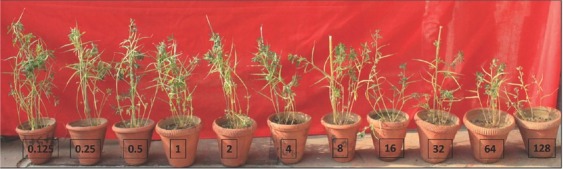
Effect of increasing nematode population densities (from 0.125 on the left to 128 J2s g^-1^ soil on the right) of *M. javanica* on the growth of fenugreek cv. UM-202, showing a reduction in plant growth. Symptoms of nematode attack (a marked reduction of plant growth) were evident at the P_i_ level of 8 J2s g^-1^ soil. However, the tolerance limits (T) of fenugreek plant shoot length were 1.3 J2s g^-1^ soil.

**Figure 3:**
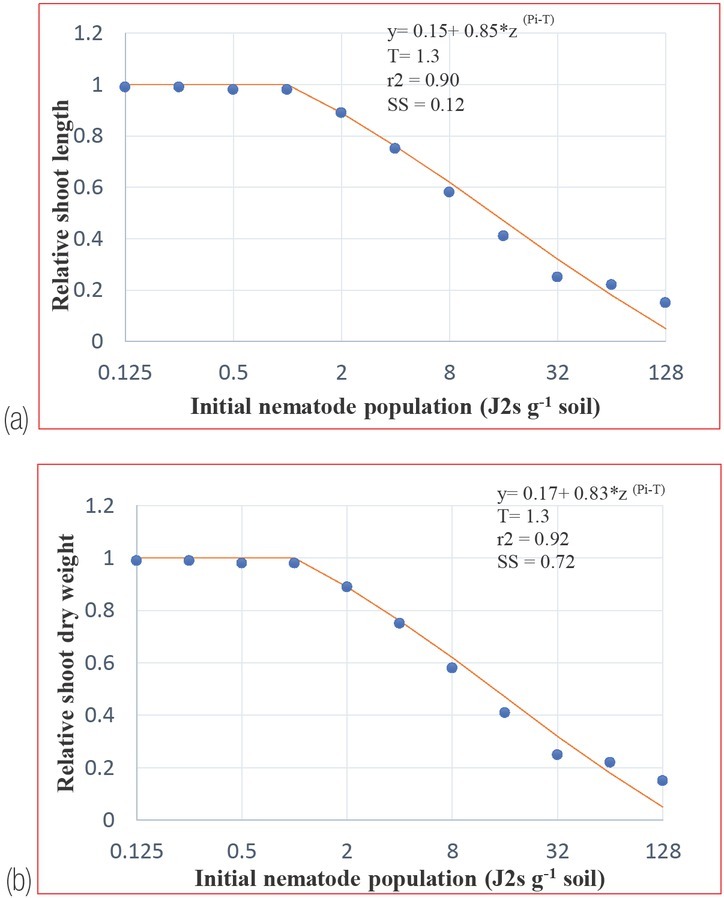
Relationship between initial population densities (Pi) of *M. javanica* and relative shoot length (A) and relative shoot dry weights (B) of fenugreek cv. UM-202, grown in pots under glasshouse conditions for 90 days. Each point represents the average of four replicated plants. Lines represent the predicted function calculated by fitting the Seinhorst model to data using the SeinFit program. Statistics for fitted models of shoot length and shoot dry weight were R^2^ = 0.90, sum of squares (SS) = 0.12; and R^2^ = 0.92, SS = 0.072, respectively.

*The relationship between the initial population density of M. javanica and the growth of fenugreek cv. UM202 under glasshouse conditions*: The initial population densities of the nematode used in this study had a negative impact on the development of fenugreek plants (Figs. 2). The relationships between initial nematode population density and shoot length and shoot dry weight were appropriately defined by the Seinhorst equation (Fig. 3A,B). Symptoms of nematode infection, i.e., stunting and yellowing and decline in plant shoot growth, were evident at 30 days after inoculation with an initial population density of 8 J2s g^-1^ soil. The fenugreek tolerance limits (T) to *M. javanica* was 1.3 J2s g^-1^ soil for shoot length and shoot dry weight, respectively (Figs. 2A,B). The relative values (m) for shoot length and dry weight were found to be 0.98 and 0.97, respectively, at the tolerance limit. The pathological parameters such as root-knot index (RKI), final nematode population (Pf) and reproduction factor (RF) were 2.9, 41.50 and 25.7, respectively at 90 days (Table 1). The minimum relative value (m) for shoot length and shoot dry weights were 0.15 and 0.17 at P_i_ = 128 J2s g^-1^ soil, respectively. The maximum reproduction rate (RF) of *M. javanica* was 31.60 at a moderate P_i_ of 2 J2s g^-1^ soil. In general, the reproduction rate decreased as P_i_ increased, and the highest final population density (P_f_ = 136.80) was found in plants inoculated with P_i_ of 8 J2s g^-1^ soil (Table 1). Correspondingly, the severity of root-knot of fenugreek was lowest at low P_i_ and highest (5.0) from P_i_ = 32 J2s g^-1^ soil onwards.

**Table 1:** **Relationship between the initial population of *Meloidogyne javanica* and root-knot index, final population density, reproduction factor in fenugreek cv***^.^* **UM-202, 90 days after inoculation**.

Initial Nematode Population (P_i_) (g^-1^ soil)	Nematode Population (P) per pot	Root-knot Index (RKI)	Final Nematode Population (g^-1^ soil) (P_f_)	Reproduction Factor (RF= P_f_ / P_i_)
0	0	0.0 ± 0.00^f^	0.0 ± 0.00^i^	0.00 ± 0.00^f^
0.125	125	0.6 ± 0.24^e^	1.20 ± 0.20^i^	9.60 ± 1.60^d^
0.250	250	1.4 ± 0.24^d^	2.80 ± 0.48^i^	11.20 ± 1.95^cd^
0.5	500	1.8 ± 0.20^d^	9.60 ± 1.32^h^	19.20 ± 2.65^b^
1	1000	2.4 ± 0.24^c^	19.80 ± 0.66^g^	19.80 ± 0.66^b^
2	2000	3.4 ± 0.24^b^	63.20 ± 1.98^d^	31.60 ± 0.99^a^
4	4000	4.4 ± 0.24^a^	52.40 ± 1.83^e^	13.10 ± 0.45^c^
8	8000	4.6 ± 0.24^a^	136.80 ± 1.98^a^	17.09 ± 0.49^b^
16	16000	4.8 ± 0.20^a^	92.60 ± 1.501^b^	5.78 ± 0.10^e^
32	32000	5.0 ± 0.00^a^	39.20 ± 1.82^f^	1.22 ± 0.05^f^
64	64000	5.0 ± 0.00^a^	55.00 ± 1.58^e^	0.85 ± 0.02^f^
128	128000	5.0 ± 0.00^a^	71.00 ± 3.75^c^	0.55 ± 0.02^f^
ANOVA TEST				
*df*		11	11	11
*SS*		192.8	97500	4252
*MS*		17.52	8864	386.5
*F*		95.6	592.2	293.6
*P*		0.01	0.02	0.02

Data present the mean ± standard deviation. Data are the averages of two trials, each with four replicate plants per treatment. The same letters within columns are not significantly different according to Duncan’s Multiple Range Test (DMRT) at *P*≤0.05. Whereas; SS (Sum of square); MS (Mean square); df (Degree of freedom); *F* (F-value); *P* (significant value)

## Discussion

Knowledge of relevant principles, together with the ability to estimate damage functions under particular circumstances for different crops, are basic requirements for determining nematode threshold values which are required for predicting crop losses caused by nematodes and selecting possible management strategies (Ravichandra, 2014). However, the presence of PPNs in the soil does not guarantee crop destruction or yield decline, because a nematode population may persist below the damage threshold level for a particular field (Talwana et al., 2016; Sasanelli et al., 2021). Many researchers have documented increases in nematode populations and consequent losses in crop growth and production, or other indications of adverse effects, which corroborate these findings (Vovlas et al., 2008; Kayani et al., 2017). As a result, even if nematode population density in the field is low, the issue cannot be ignored.

In this investigation, the intensity of root-knot index and the severe reduction of growth parameters of the fenugreek plant with increasing initial inoculum densities in the soil have validated that *M. javanica* has a high destructive capability and reproductive capacity in this crop. In this study, the establishment and invasive habits of *M. javanica* reported in fenugreek were analogous to those described in other vegetable crops (Vovlas et al., 2005). Plant emergence of fenugreek was not affected by initial population densities (P_i_) of *M. javanica*, but later growth of the plants was adversely affected. The pathological relationship of *M. javanica* inoculum on fenugreek was explained clearly by the Seinhorst function. The predicted tolerance limit of this plant to the RKN using this model was 1.3 J2s g^-1^ soil (corresponding to 1300 J2s kg^-1^ soil) for shoot length and dry weight. Compared to uninoculated control plants of fenugreek, an initial population density of *M. javanica* over 32 J2s g^-1^ soil can reduce shoot length by up to 75%, indicating the significant vulnerability of fenugreek cv. UM202 to *M. javanica*, which was further corroborated by the high reproduction rate (31.6). The reproduction factor, expressed as an RF, was the best parameter for measuring nematode infection. However, as we increased the initial nematode population (Pi) above the damage thresholds, the final nematode population and reproduction factor were decreased at all higher inoculation doses of above 2 J2s g^-1^ soil of Pi. At the same time, root-knot indexes (RKI) were increased at all higher inoculation doses of Pi. The maximum RKI was 5.0, noted at 32 J2s g^-1^ soil. Previous studies have not evaluated the pre-plant inoculum thresholds for *M. javanica* on fenugreek. However, in other crops, pre-plant inoculum thresholds for RKNs were calculated using the Seinhorst equation. The threshold level of *M. javanica* in potatoes for fresh shoot weight and height was estimated at 0·50 and 0·64 eggs and J2s g^-1^ soil, respectively (Vovlas et al., 2005). Similarly, the threshold level of *Meloidogyne* spp. for the fresh shoot and total plant weight of spinach plants was as low as 0·25 and 0·5 eggs and J2s per cm^3^ soil, respectively (Di-Vito et al., 2005).

The stunted growth of fenugreek with a large *M. javanica* inoculum could be attributed to root penetration and migration of numerous nematode juveniles during the early phases of plant growth. When the level of nematode inoculum was increased, the number of root galls increased. The gradual reduction in plant development caused by an increase in *Meloidogyn*e spp. inoculum levels has been noted in various crops (Waghmare et al., 2022; Siengchin et al., 2020; Dhurwey et al., 2019).

Despite fenugreek’s exceptional nutritional and medicinal properties, only a few studies have been completed on its management and estimation of its tolerance limit against RKNs. According to previous studies, nematode density in the soil directly impacts the host’s physiological response, and the consequences would enhance as nematode density increases. Our experiment evaluated the nematode pathogenicity and estimated the threshold level for fenugreek plants. The results revealed that the initial inoculum level of 1.3 J2s g^-1^ (1300 J2s kg^-1^) soil significantly reduced the growth attributes, namely, the shoot length and shoot dry weight. The maximum reduction was recorded at the inoculum level of 128 J2s g^-1^ soil compared to the uninoculated control. However, the biotic and abiotic factors influence the nematode dispersion (Hamza et al., 2017). These limitations are inherent; therefore, performance improvements require more research on different fenugreek varieties to varying seasons in field conditions. It is also imperative to develop longterm management strategies for RKNs by precisely measuring their population density and prevalence in the field. To the best of our knowledge, there is no specific report on the *M. javanica* threshold level on fenugreek. These findings contribute to developing field management measures to keep *M. javanica* populations below damaging levels in infested areas.
